# Effects of a physical exercise programme on bodyweight, body condition score and chest, abdominal and thigh circumferences in dogs

**DOI:** 10.1186/s12917-024-04135-3

**Published:** 2024-07-06

**Authors:** Josefin Söder, Erika Roman, Johanna Berndtsson, Katrin Lindroth, Anna Bergh

**Affiliations:** 1 Department of Clinical Sciences, Faculty of Veterinary Medicine and Animal Science, Swedish University of Agricultural Sciences, Box 7054, Uppsala, 75007 Sweden; 2 Department of Animal Biosciences, Faculty of Veterinary Medicine and Animal Science, Swedish University of Agricultural Sciences, Box 7023, Uppsala, 75007 Sweden; 3 Department of Pharmaceutical Biosciences, Faculty of Pharmacy, Uppsala University, Box 591, Uppsala, 75124 Sweden

**Keywords:** BCS, Body condition score assessment, Canine, Morphometric measurements, Morphometric ratios, Obesity, Overweight, Physical activity

## Abstract

**Background:**

Research on the effects of physical exercise on canine body composition is limited. The aim of this study was to investigate the effects of a physical exercise programme on bodyweight, body condition score (BCS) and chest, abdominal and thigh circumferences in dogs. Twenty-one healthy dogs of different breeds exercised together with their owners during an eight-week programme consisting of jogging and strength exercises. Standardised measurements were performed in triplicates with a measuring tape on standing dogs. Chest circumference was measured at three anatomical locations, abdomen at two and thigh at one. Data on bodyweight, BCS (9-point scale) and circumferences were analysed with mixed model repeated measures analyses to evaluate changes after the programme and effects of target distance.

**Results:**

Seven dog owners choose a target distance of 2 km and 14 owners choose 5–10 km. Mean BCS decreased (*P* = 0.007) after the programme (5.1 ± 0.9 vs. 4.7 ± 0.6) but there was no effect of target distance. Almost all chest and abdominal circumference measurements decreased (*P* ≤ 0.007) with the 2 km group driving the reduction in chest circumference and the 5–10 km group driving the reduction in abdominal circumference. In contrast, thigh circumference (28.8 ± 0.4 vs. 30.2 ± 0.4) increased (*P* = 0.007) while bodyweight was maintained. There were positive correlations between BCS and abdominal/chest ratios before and after the programme (Pearson correlation; R square ≤ 0.43, *P* ≤ 0.0012) but the mean ratio remained constant.

**Conclusions:**

Results indicated a redistribution between total body fat and muscle mass in body composition of normal weight to slightly overweight dogs after the physical exercise programme. The use of bodyweight alone was not a reliable evaluation method to complement the BCS assessment. However, repeated measurements of chest, abdominal and thigh circumference might aid in the assessment of body composition in dogs performing physical exercise. Further research should include a control group and objective evaluations of total body fat and lean mass, in order to investigate the effectiveness of physical exercise as a freestanding method for decreasing BCS and increasing muscle mass in overweight dogs.

**Supplementary Information:**

The online version contains supplementary material available at 10.1186/s12917-024-04135-3.

## Background

The importance of physical activity in humans has been identified in almost all sustainable development goals [1] but little is known of physical demands for dogs to remain healthy. Insufficient physical activity has been identified as an emerging cause for obesity development in companion dogs [2]. Young dogs tend to be more physically active than older dogs, large dogs more active than smaller dogs [3] and obese dogs spend less time in vigorous physical activity than normal weight dogs [4]. Previous interventions of physical activity in dogs have focused mostly on individuals intended to lose weight. Few studies so far have evaluated the effect of physical exercise solely or the combined effects of physical exercise and caloric restrictions on canine body composition [5–7]. Dog owners might be more physically active than non-dog owners [8, 9], but despite this, up to 70% of dog owners in the USA were considered insufficiently physically active and 40% walked their dogs for less than ten minutes at the time [9]. A meta-analysis concluded that owners in general walk their dogs on average 160 min per week [8]. However, dog owners in Canada that claimed not walking their dogs regularly walked their dogs only 40 min per week [10]. A large European study showed that lack of interest in performing physical activity by the owner increased overweight in both the owner and the dog [11], highlighting the importance of involving dog owners in canine intervention studies of physical activity.

Overweight in dogs is a growing health problem, in Sweden [12, 13] as in many other countries worldwide [11, 14–16]. Especially dog owners tend to underestimate the body condition of their dogs [12, 17–19]. Assessment of overweight in dogs is performed by the use of a body condition score (BCS) system, a well-established semi-subjective method with good inter- and intra-reliability but the technique requires training [12, 20]. The 9-point BCS system is widely used by veterinary healthcare personnel in the clinic [21] as the scale is based on visual and palpable hallmarks that correlate primarily to the total body fat of the dog [22]. The scale differentiates slightly overweight dogs (BCS 6) from overweight (BCS 7) and obese dogs (BCS 8–9) [22]. However, veterinary health care personnel need objective methods suitable for repeated evaluations that can differentiate between fat and muscle mass, for evaluation of overweight dogs undergoing caloric restrictions and/or for dogs undergoing physical exercise programmes. Therefore, there is a need for new objective methods that may complement the clinical BCS assessment of dogs [23], such as morphometric body measurements.

Lean body mass, in which muscle mass is included, is reduced when overweight dogs lose weight [7, 24]. One study is however indicating that lean mass may be preserved if physical activity is added to a weight-reduction programme [7]. In another study, dogs that were more physically active had a higher energy intake while maintaining weight-loss goals, compared to less active dogs [25]. For dogs intended to adjust their overall body composition, it is therefore important to include evaluation methods that are capable of detecting potential redistributions between fat and muscle mass [23]. Objective or semi-objective clinical methods used today for evaluation of canine body composition are trunk and limb circumference measurements with measuring tape [5, 6, 26–28], ultrasound measurement of subcutaneous fat thickness [29, 30], bioelectric impedance measurement of total body fat [31] or measurement of skin fold thickness with callipers [29]. Some of these methods, e.g. chest and abdominal circumference measurements [26], ultrasound measurement in the lumbar region and skin fold thickness [29] have earlier been associated to BCS of dogs. The most commonly used clinical evaluation method to complement a BCS assessment is the recording of bodyweight. Bodyweight has been claimed to correlate to changes in BCS during weight reductions and actual bodyweight is combined with BCS to calculate an ideal target weight in overweight or obese dogs [32]. However, the use of bodyweight alone to assess if a dog of a certain breed is underweight, of ideal weight or overweight is not recommended [33]. Although breed clubs state standard weights for breeds, individual dogs within a breed differ in size, body composition and configuration. In addition, breed mean weights may develop over longer periods [34]. Another drawback with recording of bodyweight as an evaluation method is that it cannot distinguish between fat and lean body mass [23], leading to the necessity of adding more refined evaluation techniques.

Generally, overall fat and lean body mass can be evaluated by advanced direct and objective methods such as dual x-ray absorptiometry (DEXA), which is a precise but costly method that requires sedation or anaesthesia of the dog [23]. Indirect methods for clinical assessment of muscle mass in dogs, other than palpation and limb circumference measurements of e.g. the thigh [27, 28, 35], are scarce. Not all evaluations methods or anatomical locations for tape circumference measurements have been validated for use in dogs, and the use or non-use of a dynamometer in order to standardise the force applied with measuring tapes differ between studies [5, 6, 26–28]. The 9-point BCS scale is specified on hallmarks for fat mass, and hallmarks for muscle loss is only included in markedly underweight dogs (BCS 1–2) [22], although muscle loss could also appear in i.e. obese dogs (BCS 8–9). In addition, neither the BCS system [22] nor the muscle condition score (MCS) system [35] for dogs include evaluation of a muscle mass above average. A prioritized question is to investigate whether the redistribution of lean and fat mass of dogs is affected in the same way by physical exercise as in humans [36]. In this context, circumference measurements of trunk and limbs might be helpful. The aim of this study was therefore to investigate how a joint physical exercise programme for dog owners and dogs, affected bodyweight, BCS and additional chest, abdominal and thigh circumference measurements in healthy dogs with a mean baseline body condition of normal weight to slight overweight.

## Methods

All clinical evaluations were conducted at the Swedish University of Agricultural Sciences, Uppsala, Sweden during August to October 2021. No data from dog owners are presented in this publication.

### Recruitment of study population

Dog owners were invited to participate in a pilot study with a joint physical exercise programme together with their dogs. They voluntarily signed up for the study through an internet-based application form distributed through the websites of the Swedish Working Dog Association and of the Swedish University of Agricultural Sciences. Participants were included on a non-randomized basis based on the following inclusion and exclusion criteria for dog owners and dogs: The inclusion criteria for owners were age ≥ 18 years and in physical and mental condition allowing participation at the minimum level in the physical exercise programme. The inclusion criteria for participating dogs were age ≥ 1 year and in physical condition allowing participation at the minimum level in the programme. Exclusion criteria for dogs included known systemic or orthopaedic disease that could entail a risk when participating in the study or known aggressiveness or timidity that could affect the ability to be evaluated by researchers. All dogs participated in the study together with their owner or handler. Sample size calculation on beforehand was not possible as this, to our knowledge, was the first study of its kind.

In total 35 dog owners with their respective dog were recruited in the study. Before the initial sample collection and start of the programme, six owners withdraw their participation and an additional seven owners withdraw their participation during the physical exercise programme. One additional dog was excluded due to its temperament that did not allow the measurement procedures performed by the researchers. Due to human error, only eleven dogs became evaluated for the most cranial chest circumference at the basal measurements, which precluded grouped analyses for that location.

### Physical exercise programme

Dog owners and dogs participated in an eight-week joint outdoor physical exercise programme designed by the Swedish Working Dog Association [37]. The programme included jogging by the owners with the dog on the side and circuit training sessions for dog owners and dogs performed together. Dog owners individually selected a target jogging distance of 2, 5, 7.5–10 km, adjusted to both owners´ and dogs´ basal fitness status, with the goal to complete the whole target distance jogging at the end of the eight weeks. Due to ethical reasons, e.g. increased risk for injury, it was not possible to randomize the assignment of target distances to dog owners and dogs or to force all participant into the same goal distance. Jogging sessions were performed twice a week, except for participants that chose the longest target distance (10 km), who performed three jogging sessions per week to enable target goal fulfilment. Distance of jogging and intensity of speed were gradually increased throughout the eight weeks for all target distances according to the programme protocol [37]. Circuit training exercises designed for joint performance by dog owners and their dogs were conducted once a week for all participants. The circuit training had four different levels of performance, from lighter to heavier exercises, to match the different target distances that the owners had chosen. Six different exercises were performed at each session; four strength exercises focused on leg/hind leg, arms/foreleg, core and neck muscles and two exercises focused on agility and explosive speed [38]. Time per exercise was gradually increased from 30 to 60 s during the programme. The physical exercise programme included no caloric restriction for participating dogs. Thus, only effects of physical exercise solely on body composition were investigated.

### Clinical data collection

The dogs underwent clinical examinations twice, before and after the physical exercise programme, by the same veterinarian and with specific focus on orthopaedic status. The clinical examination included observation of general appearance, evaluation of skin, mucous membranes and cortical refill time, auscultation of heart and lungs and abdominal palpation. Regarding the locomotor apparatus, lameness was assessed visually, palpation of the back and joints were performed as well as extension and flexion of the extremities.

All clinical data were collected twice by the same evaluators in connection to the clinical examinations. Evaluators were blinded to the target distance (2, 5, 7.5–10 km) selected by the participants and were in addition blinded to scores and measurements collected before the physical exercise program during the second evaluation after the programme. All dogs were acquainted with the research setting and thereafter put on an examination table. If needed, the dogs were corrected so that they stood squarely with the paws symmetrically.

#### Bodyweight and body condition score

Bodyweight was measured to the nearest hg with a Kruuse Scale 250 digital veterinary scale (Jørgen Kruuse A/S Langeskov, Denmark). The same veterinarian with specific expertise assessed the BCS of all dogs. Body condition assessments was performed according to guidelines from the World Small Animal Veterinary Association (WSAVA) based on the 9-point BCS scale validated by Laflamme (1997) [22] including observation and palpation of areas over the ribs, waist and abdominal line. According to this scale, a BCS of 1–3 represents underweight, 4–5 ideal weight, 6 slight overweight, 7 overweight and 8–9 represent obesity. Bodyweight and BCS assessments were collected in single measurements.

#### Chest and abdominal circumferences

**Fig. 1:**
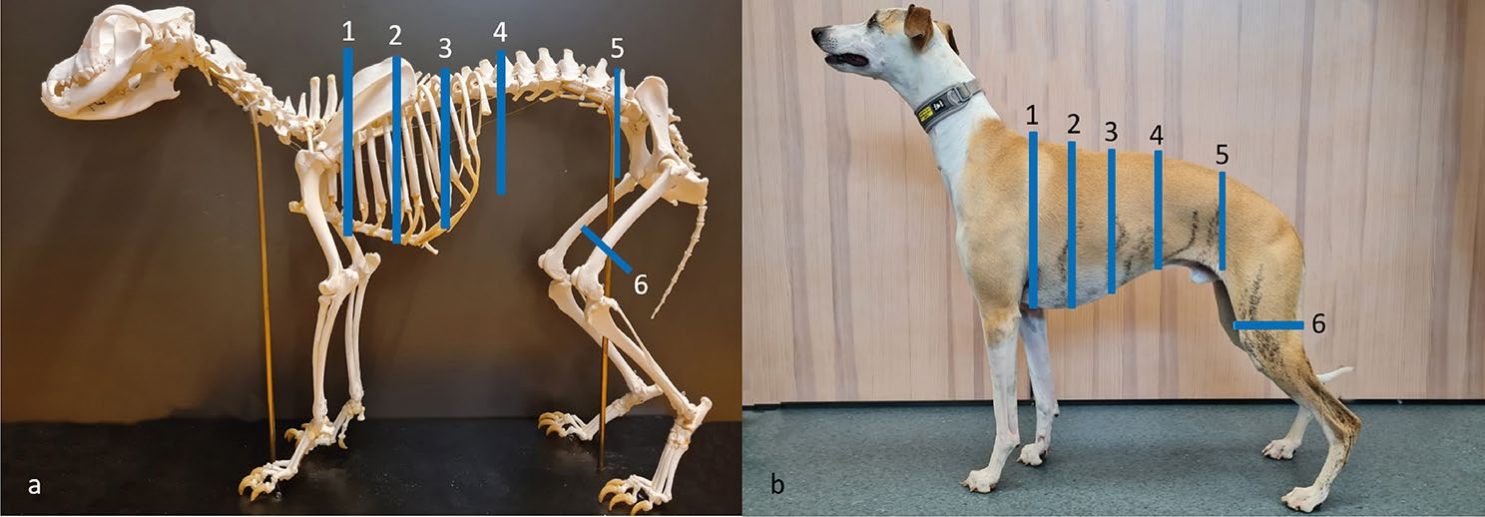
Anatomical locations for tape circumference measurements over the chest, abdomen and thigh. All measurements were performed in triplicates without a dynamometer for all locations except for the thigh where a spring tension dynamometer was used. 1: Cranial chest (in the axilla), 2: Widest chest (visually assessed from above), 3: 9:th rib (over the 9:th rib), 4: Cranial abdomen (caudal of the last rib), 5: Caudal abdomen (cranial of ileum) and 6: Thigh (70% of femur). **a**) Dog skeleton from the historic anatomical theatre, Swedish University of Agricultural Sciences. **b**) Whippet participating in the physical exercise programme, photo from baseline. Photos: Josefin Söder

Figure 1 shows markings of exact anatomical locations over the chest and abdomen used for circumference measurements. The circumferences of the chest were measured at three locations; directly caudal of the elbow in the axilla, at the widest location evaluated visually from above and over the 9:th rib. The circumferences of the abdomen were measured at two locations; directly caudal of the last rib and directly cranial of the cranial crest of ileum at the pelvic bone. Each location was measured in triplicates (in millimetres) with a measuring tape without a spring tension dynamometer.

#### Thigh circumference

Thigh measurement was included in the study as an indirect measure of muscle mass [39, 40]. The circumference of the thigh was measured at 70% of the length of the femur measured from the trochanter major´s proximal tip to the middle of the lateral fabella [28], see Fig. 1. The location was measured in triplicates with a measuring tape equipped with a mechanic spring tension dynamometer. The measuring tape was pulled horizontally (90^o^ angle to the femoral bone), with 0.3 kg (3 N) force. A person, other than the person using the measuring tape, read and recorded the measured value (in millimetres).

### Data processing and statistical analyses

**Fig. 2:**
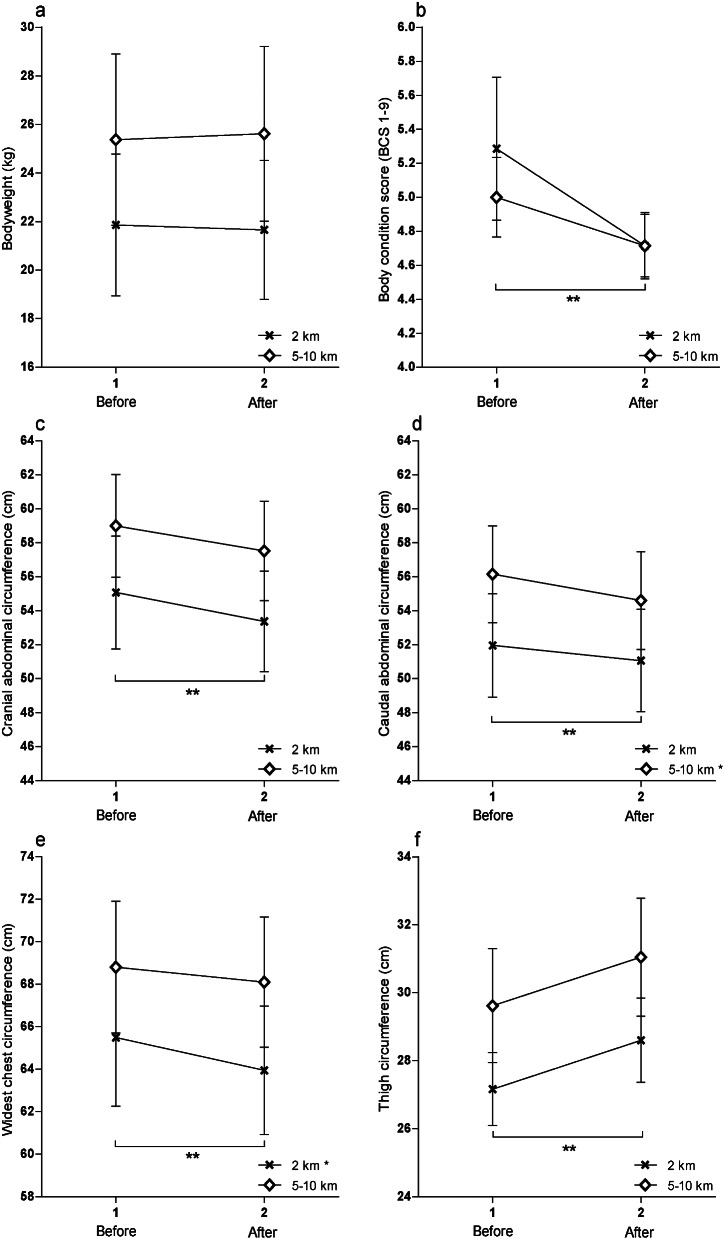
Circumference measurements of all 21 participating dogs before and after the physical exercise programme. Data is visualized by the effect of the selected target distance (2 km or 5–10 km) on six outcomes (**a**-**f**). Data were analysed with mixed model repeated measurement analyses. Significant results are marked with asterisks (* ≤ 0.05 and ** ≤ 0.01)

Microsoft Excel, SAS (SAS 9.4 Institute Inc., Cary, NC), and GraphPad Prism (GraphPad Prism 5.0 San Diego, CA) were used for data treatment, statistical analyses and graphical presentation of results. Data from participants who withdrawn before or during the study were excluded. For data collected in triplicates, the mean of the three measurements of each location and dog was used in the repeated measurement analyses. Testing of normal distribution was performed with visual assessment of residuals in the mixed model repeated measures analyses in SAS and D´Agostino and Pearson omnibus normality tests were used for evaluation of all other data prior to analyses. The threshold for statistical significance was set to *P* < 0.05 in all analyses. Results are presented as mean value ± standard deviation (SD), except for visualization of data in Fig. 2 and for precision of measurements in Table 2, where standard error of mean (SEM) are used.

#### Mixed model repeated measures analyses

All data, except for the most cranial chest circumference (due to missing data), were evaluated by mixed model repeated measures analyses in SAS [41–43] for comparisons of the time points before and after the exercise programme with regard to the chosen target distance. For the mixed model repeated measures analyses, data were divided into two groups based on the selected target distance of the owner (2 km and 5–10 km). Pooling of the 5, 7.5 and 10 km groups was performed due to that few owners choose the two longest distances. In the statistical model, the chosen target distance (2 km or 5–10 km) was defined as an independent variable. The mixed model repeated measures model analysed the response between time points (before and after the programme), the response between groups (2 km or 5–10 km) and pairwise comparisons before and after the physical exercise programme and between groups (interactions). The model corrected for multiple comparisons by Tukey-Kramer adjustment. For data on bodyweight, logarithmic transformation of raw data was applied in the mixed model as data was not normally distributed.

#### Paired and un-paired analyses

The cranial chest circumference and the ratio between cranial abdominal circumference and widest chest circumference were evaluated by a paired t-test for comparisons of changes before and after the physical exercise programme. Data for morphometric measurements stratified into sex (female versus male dogs) and breed size (small/medium versus large/giant dogs) were evaluated with Wilcoxon signed rank test for differences after the physical exercise programme compared to before as this data was not normally distributed. Dogs were divided into different breed sizes according to previously defined ranges based upon the baseline bodyweight of dogs before the start of the physical exercise programme [44]. Data on differences in BCS between male and female dogs at the time point before the exercise programme were not normally distributed and were evaluated with the Mann Whitney U-test.

#### Correlation analyses

Pearson correlation was used to analyse correlations between BCS and the ratio (cranial abdominal circumference/widest chest circumference) at the time points before and after the physical exercise programme. Pearson correlation was also used to analyse the correlations between BCS and all circumference measurements (cranial chest, widest chest, 9:th rib, cranial abdomen, caudal abdomen and thigh) as well as for correlation between the cranial abdominal circumference and the widest chest circumference.

## Results

### Description of dog population

A total of 21 owners and their dogs completed all parts of the study. None of the participating dogs showed deviations on the initial clinical examination that hindered participation in the physical exercise programme. Nor did the programme result in any adverse effects afterwards, related to the locomotor apparatus. No dogs showed visual signs of lameness at any of the two clinical examinations, either before or after the programme. At the clinical examination before the exercise programme, 13 dogs showed minor palpatory findings of the locomotor apparatus, not assessed as a contraindication for inclusion in the study. Raw data of all dogs is presented in Supplementary Material 1.

### Baseline characteristics

Table 1 shows the baseline characteristics of participating dogs at the time point before the exercise programme. The dogs were of a variety of small, medium, large and giant sized breeds and a total of 15 different breeds plus mixed breeds, were represented. The breeds were; mixed breed (*n* = 2), Flatcoated Retriever (*n* = 2), Lagotto Romagnolo (*n* = 2), Schapendoes (*n* = 2), German Shepherd (*n* = 2), Bull Mastiff (*n* = 1), Golden Retriever (*n* = 1), Hovawart (*n* = 1), Icelandic Sheepdog (*n* = 1), Malinois (*n* = 1), Medium Poodle (*n* = 1), Siberian Husky (*n* = 1), Småland Hound Spinone (*n* = 1), Staffordshire Bull Terrier (*n* = 1), Welsh Springer Spaniel (*n* = 1) and Whippet (*n* = 1). Female dogs had significantly higher mean BCS than male dogs (*P* = 0.04) before the start of the physical exercise programme, but large/giant sized dogs did not differ from small/medium sized dogs in mean BCS (*P* = 0.62; Table 1).

**Table 1:** Baseline characteristics of participating dogs (*n* = 21) before the start of the physical exercise programme

	Whole dog cohort (*n* = 21)	
*Parameter*	*Mean ± SD*	
**Age (years)**	5 *±* 3	
**Bodyweight (kg)**	24.2 ± 11.3	
**BCS (scale 1–9)**	5.1 ± 0.9	
**Sex**	*Number (of which neutered or spayed)*	
Male	10 (3)	
Female	11 (4)	
**Target distance (km)**	*Number (M/F)*	*Number (SM/LG)*
2	7 (3 M/4F)	7 (3 M/4L)
5	9 (7 M/2F)	9 (4 SM/5LG)
7.5	4 (4 F)	4 (4 M)
10	1 (1 F)	1 (1 M)
**BCS (scale 1–9)**	*Number (M/F)*	*Number (SM/LG)*
1–2 (underweight)	0	0
3 (slight underweight)	1 (1 F)	1 (1 M)
4–5 (normal weight)	13 (9 M/4F)	13 (7 SM/6LG)
6 (slight overweight)	6 (1 M/5F)	6 (3 M/3L)
7 (overweight)	1 (1 F)	1 (1 M)
8–9 (obese)	0	0
**BCS (scale 1–9)**	*Mean ± SD*	
Male	4.7 ± 0.7*	
Female	5.5 ± 1.0*	
2 km group	5.3 *±* 1.1	
5–10 km group	5.0 *±* 0.9	
Small/Medium sized^†^	5.0 ± 1.1	
Large/Giant sized^††^	5.22 ± 0.6	

BCS: body condition score, M: Male, F: Female, kg: kilograms, km: kilometre, LG: Large to giant sized dogs, SD: Standard deviation, SM: Small to medium sized dogs. Mean BCS was numerically higher in the 2 km group compared to the 5–10 km group and in Large/Giant sized dogs compared to Small/Medium sized dogs at baseline but did not differ significantly (*P* ≥ 0.62). Data that were significantly different between groups are marked with an asterisks (* *P* < 0.05). ^†^Of which one dog was small sized of < 10 kg. ^††^Of which two dogs were giant sized of > 40 kg.

### Effects of the physical exercise programme

#### Bodyweight and body condition score

**Fig. 3:**
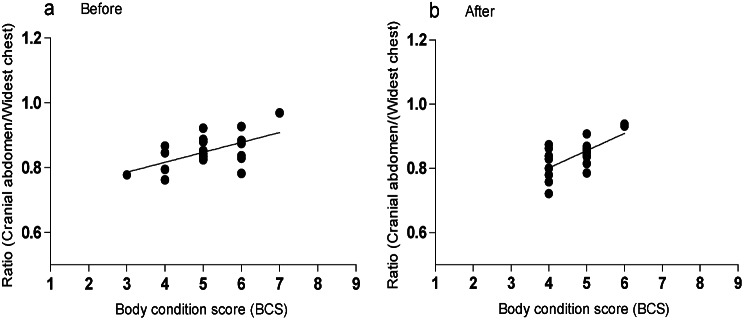
Correlation of body condition score (BCS) and morphometric ratios of all 21 participating dogs. **a**) Before the physical exercise programme and **b**) after the physical exercise programme, with each dot representing an individual dog. Pearson correlation was used for analyses of correlation between BCS and ratios (cranial abdominal circumference/widest chest circumference) at both time points (before and after the exercise programme)

Bodyweight of participating dogs did not change after (24.3 ± 11.8 kg) compared to before (24.2 ± 11.3 kg) the physical exercise programme (*P* = 0.98, Fig. 2a). Body condition score on the other hand decreased after (4.7 ± 0.6) compared to before the physical exercise programme (5.1 ± 0.9, *P* = 0.007, Fig. 2b). The significant decrease in BCS applied to all dogs and was not dependent on the chosen target distance. The dog population as a whole was transforming towards an ideal body condition of a BCS of 4–5 after the physical exercise programme, visualized by the dots illustrating individual dogs in Fig. 3.

### Abdominal circumferences

Both cranial and caudal abdominal circumferences decreased significantly, after the physical exercise programme compared to before (Table 2; Fig. 2c–d), where the reduction in the caudal abdominal circumference was driven by the 5–10 km group (Fig. 2d).

**Table 2:** Morphometric circumference measurements of all 21 participating dogs, at the time points before and after the physical exercise programme

Circumference	Before	After	*P*-value	Precision of measurements
*cm*	*Mean ± SD*	*Mean ± SD*	*< 0.05*	*Mean SEM ± SD* ^*b*^
Cranial abdomen	57.7 ± 10.5	56.1 ± 10.0	*P* = 0.004	0.20 ± 0.15
Caudal abdomen	54.8 ± 9.9	53.4 ± 9.9	*P* = 0.007	0.17 ± 0.10
Cranial chest^a^	69.8 ± 11.9	67.3 ± 11.2	*P* = 0.001	0.15 ± 0.10
Widest chest	67.7 ± 10.6	66.7 ± 10.4	*P* = 0.003	0.17 ± 0.14
9:th rib	66.4 ± 10.0	65.5 ± 10.7	*P* = 0.120	0.26 ± 0.22
Thigh	28.8 ± 5.4	30.2 ± 5.7	*P* = 0.007	0.40 ± 0.23

Cm: Centimetre, SD: Standard deviation, SEM: Standard error of mean of the triplicates. Analyses were performed with mixed model repeated measurement analyses except for data on the “cranial chest” that was analysed with a paired t-test due to missing data (^a^ analysis was based on 11 dogs). ^b^ Circumference measurements were performed before and after the physical exercise program with a measuring tape in triplicates, of which SEM was calculated. Means of SEM ± SD for all dogs are shown with the two time points (before and after the physical exercise programme) pooled.

### Chest circumferences

The cranial and widest chest circumferences both decreased significantly after the physical exercise programme (Table 2), where the reduction in the widest chest circumference was driven by the 2 km group (Fig. 2e). The most caudal chest circumference, measured over the 9:th rib, did not change after the physical exercise programme (Table 2).

### Thigh circumference

The thigh circumference was the only circumference measure that, opposite to the other circumference measurements, increased after the physical exercise programme compared to before (Table 2; Fig. 2f).

### Different morphometric body measures stratified into sex and breed size of dogs

All dogs, independent of stratum (female or male and/or small/medium size or large/giant size) withheld a stable bodyweight (Supplementary Material 2). Male and female dogs as well as small/medium and large/giant sized dogs all showed numerically decreased BCS after the exercise programme compared to before, but the decrease was significant only for large/giant sized dogs (Supplementary Material 2). Male and female dogs as well as small/medium and large/giant sized dogs showed numerically decreased abdominal circumferences after the exercise programme compared to before. The decrease was significant for males and/or large/giant sized dogs regarding the cranial abdomen and significant for females and/or small/medium sized dogs for the caudal abdomen (Supplementary Material 2). In males and/or in large/giant sized dogs the chest circumference over the 9:th rib did not change. All other strata showed numerically decreased chest circumferences (both regarding the widest chest and over the 9:th rib), with the decrease being significant for females and/or large/giant sized dogs regarding the widest chest and significant for females and/or small/medium sized dogs over the 9:th rib (Supplementary Material 2). All strata showed numerically increased thigh circumferences after the exercise programme compared to before, with the increase being significant for females and/or small/medium sized dogs (Supplementary Material 2).

### Correlation of body condition score and morphometric measurements

There was no correlation (*P* ≥ 0.09) between BCS and any of the circumference measurements (cranial chest, widest chest, 9:th rib, cranial abdomen, caudal abdomen or thigh). There was a positive correlation between the cranial abdominal circumference and the widest chest circumference (*P* < 0.0001, R square 0.90).

The mean ± SD of the morphometric ratio derived from the cranial abdominal circumference divided by the widest chest circumference was equal (*P* = 0.15) before (0.85 ± 0.05) compared to after (0.84 ± 0.05) the physical exercise programme. There was a positive correlation (R square 0.32–0.43) between this ratio and the assessed BCS, both before (Fig. 3a) and after (Fig. 3b) the exercise programme (*P* ≤ 0.001). The mean ± SD of the morphometric ratio derived from the caudal abdominal circumference divided by the widest chest circumference was equal (*P* = 0.11) before (0.81 ± 0.05) compared to after (0.80 ± 0.05) the physical exercise programme. There was a positive correlation (R square 0.26–0.27) between this ratio and the assessed BCS, at the time points both before and after the physical exercise programme (*P* ≤ 0.017).

## Discussion

In this study, normal weight to slightly overweight dogs that participated in a physical exercise programme, freestanding from caloric restriction, maintained a stable bodyweight. However, BCS, chest and abdominal circumferences decreased while the abdominal/chest ratio remained unchanged. In contrast, the thigh circumference increased. The results are thus pointing towards a redistribution between total body fat and muscle mass as an effect of the physical exercise programme.

### Effects of physical exercise on canine body composition

Results showed decreased BCS, maintenance of a stable bodyweight and increased thigh circumference after the physical exercise programme. After the programme, 19 out of 21 dogs were assed to be of ideal weight (BCS 4–5), compared to 13 dogs before the programme. Previous studies with the exact same study design, using a physical exercise programme solely, freestanding from the use of caloric restriction as the current study, are however lacking. One study by Vitger et al. (2016) that used objective methods for evaluation of fat and lean body mass, added physical activity to one of two groups of overweight dogs in a caloric restriction intervention study [7]. Results indicated that by increasing physical activity by 13% compared to baseline, lean body mass was preserved during weight loss, while lean body mass instead was lost when physical activity was not added. In a study by Chauvet et al. (2011), obese dogs that combined physical exercise with a caloric restriction significantly lost body weight [6]. In a study of overweight dogs by Chapman et al. (2019), bodyweight decreased in a calorie-restricted group but not in a group performing exercise solely [5]. An inclusion of objective evaluation methods or limb circumferences for assessment of overall body composition in those studies [5, 6] could perhaps have enabled detection of redistributions between body fat and lean mass that might have been present. The indication of increased tight muscle mass in the current study is further supported by the fact that dogs exercising the longer distances (5–10 km) was the group having the highest impact on the reduction in the mean abdominal circumference. It is thus possible that dogs jogging the longer distances could have improved their abdominal muscle mass in addition to their increased thigh circumference. Increased physical activity might have led to increased strength of the abdominal muscles resulting in an increased abdominal tuck [22] thereby decreasing the abdominal circumference. Significantly increased thigh circumference has previously been reported in healthy dogs jogging on a treadmill twice a week for twelve weeks [45] where the increased circumference has been proposed to indirectly associate to increased muscle mass [46]. Only obese dogs develop fat deposits over the legs [22]. No obese dogs participated in the current study and therefore, intramuscular or subcutaneous fat over the limbs should not have influenced thigh circumferences to any major extent.

A higher starting BCS in a stratum (male or female and/or small/medium or large/giant size) indicated an increased number of significant changes in morphometric body measures (Supplementary Material 2). The results indicated that overweightness, rather than sex and/or breed size, influenced the changes of the different measures in this study. In addition, dogs with lower starting BCS and/or a bodyweight of > 25 kg did not show a decrease in the most caudal chest circumference, an anatomical location previously shown prone to development of fat deposits [30, 47]. However, all dogs, regardless of sex or size, numerically increased their thigh circumference after the exercise programme. The effect of dog intrinsic factors merits further investigation, as it is possible that dogs of different sex or size, independently of body conditions status, may respond differently to physical excise with regard to redistributions of fat and muscle mass.

### Evaluation methods to complement BCS assessments in dogs performing physical exercise

Our data show that the use of bodyweight alone is not a reliable evaluation method to complement BCS assessments in dogs performing physical exercise. Especially, if dog owners are instructed to reduce dogs´ BCS by increased physical activity, a positive reduction in BCS might be missed if only the bodyweight is recorded. Here, recordings of the actual chest and abdominal circumferences might be a preferred evaluation method in combination with BCS assessments; that should be taught in a standardized manner [12]. Chun et al. 2019 found that the ratio of abdominal and chest circumferences correlated to BCS of Beagle dogs with a BCS of 3–8 that gained weight [26]. Equally, our results showed a significant correlation of BCS and the abdominal and chest ratio but in a cohort consisting of small to giant sized breeds. Caution should however be taken if ratios of abdominal and chest circumferences are used for repeated evaluations of body composition in dogs performing physical exercise. The ratio, as suggested by Chun et al. (2019), could not in the present study be used as a marker for a changed BCS. The ratio remained constant after the exercise programme compared to before, due to a parallel decrease in chest and abdominal circumferences. However, repeated measurements of chest, abdominal and thigh circumferences might aid the assessment of changes in body composition in dogs performing physical exercise, as those anatomical locations together may provide indirect estimations of both fat and muscle mass status.

### Anatomical locations for circumference measurements over the chest

In this study, the cranial and the widest chest circumferences both decreased significantly after the programme, along with a corresponding decrease in BCS. These results are supported by a study that compared caloric restriction with increased physical activity during eight weeks [5]. Results from that study showed that overweight dogs that performed exercise or reduced their energy intake decreased their chest circumference; even though the increased exercise did not produce weight loss itself [5]. Another study of obese dogs that received caloric restriction combined with physical exercise during 12 weeks (led walks by their owners and underwater treadmill), equally showed that the chest circumference decreased significantly together with a loss of bodyweight [6]. When dogs on the other hand increase in total body fat evaluated by DEXA [22], the BCS increases. This is clinically evaluated by palpation, trunk circumference measurements or by ultrasound measurement of subcutaneous fat, particularly over the chest and lumbar area [22, 26, 30, 47]. In the current study, the group of owners that choose the shortest target distance of 2 km had dogs with numerically higher BCS before starting the physical exercise programme than the dogs´ of owners that choose longer target distances. Interestingly, those dogs with numerically higher BCS had a higher impact on the reduction in mean chest circumference, further underlining that a decrease in BCS may be supported by a decreased chest circumference. Circumference measurement over the chest is therefore a promising location to complement a BCS assessment when dogs are evaluated repeatedly, especially as clinical evaluation of the fat layer over the ribs is vital in a BCS assessment [22, 47].

Two different anatomical locations for circumference measurements of the chest are in the axilla and at the widest location assessed from above, which are promising locations for use in clinical evaluations shown in this study as in previous studies [5, 6, 26, 48]. However, the exact anatomical location of the widest chest could in fact change when dogs increase or decrease in BCS. A methodological improvement for further studies could be to clip a marking in the fur at the first evaluation to ensure that the exact location will remain for the repeated measurement. The most caudal measurement over the 9:th rib was difficult to perform in a standardized manner in the current study as the measurement tape was sliding backwards over the last ribs not attached to the sternum. In addition, this circumference measure did not decrease as the BCS decreased, but this could also be due to measurement error. Nevertheless, studies have demonstrated associations between BCS or body weight with thickness of the subcutaneous fat layer over the 9:th rib [30, 47], even though according to our data, measurements performed more cranially over the chest might better associate to changes in BCS.

### Anatomical locations for circumference measurements over the abdomen

Both the cranial and caudal abdominal circumferences decreased significantly after the physical exercise programme. A study by Chun et al. (2019) investigated Beagle dogs during a weight-gaining intervention of 16 weeks, and showed that abdominal circumference increased as BCS increased [26]. Likewise, a study of inactive cats showed a correlation of total body fat and the circumference of the cranial abdomen [49]. It should however be noted that those studies used cats that were of quite uniform size, as well as solely one dog breed, and results might therefore not extrapolate to other cohorts of dogs that differ more in size or configuration. Two other studies that investigated either effects of physical activity [5] or the combined effects of caloric restriction and physical activity [6] in overweight dogs likewise showed that abdominal circumferences deceased during the interventions. Our data support that abdominal circumference in dogs might decrease along with decreased BCS and/or during performance of physical exercise, as those factors could not be separated by the current study design. A study by Mugnier et al. (2023) found a positive correlation between BCS and subcutaneous fat thickness in the lumbar region measured with ultrasound [30]. Another study showed that neutered dogs that increased in total body fat evaluated with computed tomography, increased the subcutaneous fat layer predominantly in the lumbar area [47]. However, the abdominal circumferences measured in the lumbar region does not necessarily, as unambiguous as the chest circumference, evaluate subcutaneous fat. Circumference measurements performed in the lumbar region might in fact include subcutaneous fat and intra-abdominal fat [47], the muscle mass of iliocostalis and longissimus musculature on the back as well as representing the strength of all four layers of the abdominal muscles to tuck up the viscera. Therefore, inclusion of the abdominal circumference in the lumbar region when evaluating dogs require more in depth anatomical knowledge of the evaluator, to correctly interpret the results.

### Precision of morphometric measurements

It is worth considering that none of the dogs had their fur clipped prior to the morphometric measurements. In a study by McCarthy et al. (2018), the average difference before and after clipping the fur at a thigh circumference measurement was only 3 millimetres [28]. On the contrary, Bascuñán et al. (2016) recorded a significant difference in thigh circumference after clipping the fur in a cadaveric dog model; but the inter-variability did not improve [27]. Clipping of the fur before circumference measurements might thus be relevant under certain conditions, e.g. when dealing with repeated evaluations during seasonal fur-thickness. Another way of achieving consistent measurements especially on long- or thick haired breeds could be to use a measuring tape equipped with a dynamometer ensuring the same force to be applied at all evaluations despite fur interference [28]. Thigh circumference is recommended to be measured at 70% length of the femoral bone, with the dog in a lateral recumbent position using a measuring tape equipped with a spring tension dynamometer [28]. However, additional reliability studies of awake dogs in standing position are warranted as this position might be the most easily achieved at clinical examinations. In the present study of awake standing dogs, the intra-variability of the thigh circumference measurements might be considered quite low as obtained mean SEM ± SD (0.40 ± 0.23), was well below the intra-variability of thigh circumference measurements previously reported (1.13 ± 0.77) in awake standing dogs [27]. The thigh circumference increased significantly, and the recorded mean difference of 1.4 cm was about three times greater than the recorded mean SEM. However, considering the intra-variability of thigh circumference previously reported (> 1 cm) [27], the obtained difference after the physical exercise programme compared to before in the current study is quite near that magnitude of measurement error. Chest circumference was measured in a study by Witzel et al. (2014) over the 4:th to 6:th rib and the abdominal circumference was measured at the 5:th lumbar vertebrae [48], anatomical locations that are right in between the locations of the “cranial” and the “widest” chest and the “cranial” and “caudal” abdomen in the current study. Measurements by Witzel et al. (2014) were performed with a measuring tape without a spring tension dynamometer and intra-variability did not contribute to the total variation while inter-variability accounted for less than 1% of the total variation [48]. Likewise, in the current study, the intra-variability for chest and abdominal circumferences appears low and the locations showing significant differences had reductions that were ≥ 6 times greater than the recorded means of SEM. However, as the measurements were performed twice, before and after an intervention on the same dog, the recorded differences in outcomes should preferably be compared to two times the means of SEM. Even though, all differences recorded after the programme were at minimum ≥ 0.6 cm larger than what could be due to the measurement errors (two times the means of SEM). To our knowledge, no studies have fully evaluated chest and abdominal circumference measurements in dogs for inter- and intra-variability. In addition, the inter- and intra-variability for tape measurements on different anatomical locations on the trunk should preferably be compared with regard to measurements performed with and without a spring tension dynamometer.

### Physical exercise as a freestanding treatment of canine overweight

New evaluation methods that are objective or semi-objective are needed to complement clinical BCS assessments, as the method is semi-subjective, requires training [12, 20] and as dog owners tend to underestimate BCS of their dogs [12, 17–19]. Our results together with results from other studies [5, 6, 26], show that repeated tape measurements of trunk and limb circumferences at standardized anatomical locations could be helpful in that context. New ways of preventing and reducing overweight in dogs are also of importance in order to decrease risks for metabolic disturbances [50], chronic diseases [51] and a shortened lifespan [52, 53] in the general canine population. Our results indicate positive effects of a short-term physical exercise programme, freestanding from caloric restriction, in dogs with a baseline BCS of 3–7 in order to reach an ideal body composition of BCS 4–5. However, only one underweight dog (BCS 3) and one overweight dog (BCS 7) was represented, and the remaining dogs were predominantly of the upper range of normal weight (BCS 5) to slight overweight (BCS 6) when entering the study. No obese dogs (BCS 8–9) were represented, but these dogs might not be the first patient of choice for physical exercise in the form of jogging as a freestanding treatment, as these individuals may suffer from painful joint problems [54]. However, the muscle mass of obese dogs would probably benefit from inclusion of water based physical exercise in weight loss programmes as have been shown in overweight dogs [7].

All dogs in the present cohort showed positive development of their body condition, even at the lowest goal of jogging 2 km distance twice a week together with their owner. What level and duration of physical exercise that is needed in dogs to maintain or increase muscle mass is however not known. Dog owners that choose the lowest target distance of 2 km in the current study were probably not that physically active with their dogs on beforehand, which could perhaps explain the positive effect on body condition achieved, despite the moderate goal. It should however be noted that participating dog owners could have been unintentionally influenced by their own healthier lifestyle exercising together with their dogs in the programme and could therefore possibly have offered the dogs less food and treats than usual. However, owners stated in questionaries’ to have maintained the same feeding routines of their dogs throughout the study [55], but objective measurements of the dogs´ food intake was not performed to verify those statements. Further studies of dogs performing physical exercise as a freestanding treatment of overweight are warranted. Studies should include indirect measures of muscle mass, such as tape measurements, as well as objective evaluations of total body fat and lean mass, using e.g. DEXA verifications, in order to increase knowledge of how overall body composition in dogs is affected by physical exercise.

### Limitations and future perspectives

This study has several limitations, e.g. the lack of baseline measurements for cranial chest circumference in 10 dogs, limited total sample size of dogs and uneven group numbers. Due to ethical reasons, participants could not be randomized to a specific target distance group. Ideally, there would have been an equal number of dogs in each group, but this was not achievable despite intense recruitment. Therefore, originally planned target distance groups had to be combined during data analyses. Further research on effects of physical exercise on overall body composition in dogs should include a control group of dogs that do not perform exercise as well as objective measurements of total body fat and lean mass in addition to morphometric body measurements. Reliability studies of chest and abdominal circumference measurements, including evaluations of the suitability of using a dynamometer attached to the measuring tape in those anatomical locations, are warranted. In the current study, dog owners stated in questionnaires to significantly have increased their physical activity time [55], but all the physical activity performed might not have been joint physical exercise together with the dog. To better record both physical activity time and intensity in future studies, dogs should preferably wear some form of activity monitors. However, as dogs were not put on any caloric restriction but nevertheless decreased BCS and increased thigh circumference while maintaining a stable bodyweight, it is possible that the physical exercise of a minimum of 2 km twice a week had a positive effect on overall body composition. It is necessary for future studies to clarify the intensity of exercise required to increase or maintain muscle mass. Muscle condition status is currently not included in the 9-point BCS scale other than in underweight dogs [22], even though muscle loss could be present in all forms of normal weight, overweight and obese conditions. Preferably, a muscle condition scoring system should be developed that ranged from under to above average to better aid the interpretation of other bodily changes. Dogs of the present study were mainly of normal weight to slight overweight conditions (BCS 4–6), which are body condition scores that are common of dogs in Sweden [12, 13]. The importance of physical activity for a healthy dog-life warrants further investigations and the responsiveness to exercise in dogs of different body conditions in terms of reducing fat mass and increasing muscle mass are questions for future research.

## Conclusions

Results indicated a redistribution between total body fat and muscle mass in the body composition of normal weight to slightly overweight dogs after a physical exercise programme. The repeated morphometric measurements of dogs performing physical exercise indicated that refined methods for evaluation of canine body composition are needed to detect potential redistributions of fat and muscle mass. The use of bodyweight alone was not a reliable evaluation method to complement the BCS assessment. However, repeated measurements of chest, abdominal and thigh circumferences might aid in the assessment of changes in fat and muscle mass in the body composition of dogs performing physical exercise. Further research on effects of physical exercise on body composition in dogs should include a control group, objective evaluations of physical activity and of total body fat and lean mass in addition to morphometric body measurements. Of particular interest is to investigate the effectiveness of physical exercise as a freestanding method for decreasing BCS and increasing muscle mass in overweight dogs.

### Electronic supplementary material

Below is the link to the electronic supplementary material.

Supplementary Material 1

Supplementary Material 2

## Data Availability

The datasets supporting the conclusions in this article are included within the article and its Supplementary Materials.

## Abbreviations

BCS: Body Condition Score
DEXA: Dual X-Ray Absorptiometry
MCS: Muscle Condition Score
SD: Standard Deviation
SDG: Sustainable Development Goals
SEM: Standard Error of Mean
