# Supplementary material for: Effects of a physical exercise programme on bodyweight, body condition score and chest, abdominal and thigh circumferences in dogs
Source: BMC Vet Res. 2024 Jul 6;20:299. doi: 10.1186/s12917-024-04135-3 (PMC11227157; doi:10.1186/s12917-024-04135-3)
Supplement: Supplementary file 2 — Supplementary Material 2 [file 12917_2024_4135_MOESM2_ESM.docx]

**Additional file 2. Morphometric body measures of the 21 dogs stratified into sex and breed size.**

|  | **Sex (n = 21)** | | | | **Breed size**^∞^ **(n = 21)** | | | |
| --- | --- | --- | --- | --- | --- | --- | --- | --- |
|  | **Male** | | **Female** | | **Small/Medium**^∞∞^ | | **Large/Giant**^∞∞∞^ | |
|  | (n = 10) | | (n = 11) | | (5–25 kg, n = 12) | | (> 25 kg, n = 9) | |
| **Measurement^†^** | **Before** | **After** | **Before** | **After** | **Before** | **After** | **Before** | **After** |
|  | *Mean ± SD* | *Mean ± SD* | *Mean ± SD* | *Mean ± SD* | *Mean ± SD* | *Mean ± SD* | *Mean ± SD* | *Mean ± SD* |
| Bodyweight *(kg)* | 26.2 ± 10.8 | 26.0 ± 10.8 | 22.4 ± 12.5 | 22.7 ± 12.9 | 16.3 ± 3.6 | 16.5 ± 3.8 | 34.7 ± 9.4 | 34.7 ± 10.1 |
| BCS *(scale 1–9)* | 4.7 ± 0.7 | 4.4 ± 0.5 | 5.5 ± 1.0 | 5.0 ± 0.6 | 5.0 ± 1.1 | 4.8 ± 0.7 | 5.22 ± 0.6^*^ | 4.6 ± 0.5^*^ |
| Cranial abdomen (*cm)* | 59.0 ± 11.9^*^ | 57.0 ± 11.7^*^ | 56.5 ± 9.5 | 55.3 ± 8.7 | 51.0 ± 6.7 | 50.1 ± 6.5 | 66.7 ± 7.0^*^ | 64.4 ± 7.3^*^ |
| Caudal abdomen (*cm)* | 56.3 ± 11.5 | 55.3 ± 11.8 | 53.3 ± 8.4^*^ | 51.7 ± 7.8^*^ | 48.3 ± 6.2^*^ | 47.0 ± 5.8^*^ | 63.3 ± 6.4 | 62.0 ± 6.9 |
| Widest chest (*cm)* | 69.4 ± 10.9 | 68.9 ± 10.5 | 66.2 ± 10.6^*^ | 64.7 ± 10.5^*^ | 60.7 ± 6.5 | 60.0 ± 6.2 | 77.0 ± 6.9^*^ | 75.2 ± 7.3^*^ |
| 9:th rib (*cm)* | 67.4 ± 10.3 | 67.3 ± 11.5 | 65.4 ± 10.2^*^ | 63.9 ± 10.2^*^ | 60.2 ± 6.6^*^ | 58.6 ± 6.5^*^ | 74.6 ± 7.3 | 74.8 ± 7.3 |
| Thigh (*cm)* | 29.5 ± 6.0 | 30.8 ± 5.5 | 28.2 ± 5.1^*^ | 29.7 ± 6.0^*^ | 25.6 ± 3.3^*^ | 27.2 ± 3.7^*^ | 33.1 ± 4.5 | 34.4 ± 5.0 |

^†^ The location of the cranial chest was excluded from the stratified statistical analyses as only data from eleven dogs were available and the number was too few to be divided into different sex and breed sizes for that anatomical location.

^*^Differences after the physical exercise programme compared to before were analysed with Wilcoxon signed rank test for male and female dogs respectively, and small/medium sized dogs and large/giant sized dogs, respectively, as data were not normally distributed. Significant changes (P < 0.05) are marked with asterisks (^*^) for each paired comparison within groups.

^∞^Dogs were divided into different breed sizes according to previously defined ranges based upon the baseline bodyweight of dogs before the start of the physical exercise programme. ^∞∞^Of which one dog was small sized of < 10 kg. ^∞∞∞^Of which two dogs were giant sized of > 40 kg

After: Measurements performed after the physical exercise programme. Before: Measurements performed before the physical exercise programme, SD: Standard deviation.
